# Sentinel Lymph Node Mapping in Endometrial Cancer: Experience from a Tertiary Referral Center in Türkiye

**DOI:** 10.3390/jcm15218410

**Published:** 2026-10-29

**Authors:** Eyüp Gökhan Turmuş, Okan Oktar, Hande Esra Koca Yildirim, Baran Yeşil, Mustafa Taze, Nigar Mammadova, İzzet Özgürlük, Çiğdem Kılıç, Burak Ersak, Caner Çakır

**Affiliations:** 1 Department of Gynecologic Oncology, Dr. Abdurrahman Yurtaslan Ankara Oncology Training and Research Hospital, Ankara 06200, Türkiye; hndesra@gmail.com; 2 Department of Gynecologic Oncology, Ministry of Health Ankara Training and Research Hospital, Ankara 06230, Türkiye; oknoktar@gmail.com; 3 Department of Gynecologic Oncology, Ankara Etlik City Hospital, Faculty of Medicine, University of Health Sciences, Ankara 06170, Türkiye; baranyesil@windowslive.com (B.Y.); mustafataze@gmail.com (M.T.); mammadlin92@gmail.com (N.M.); cigdemkilic2002@gmail.com (Ç.K.); burakersak@gmail.com (B.E.); caner4084@gmail.com (C.Ç.); 4 Department of Gynecology and Obstetrics, Ankara Bilkent City Hospital, Faculty of Medicine, University of Health Sciences, Ankara 06800, Türkiye; izzet.ozgurluk@sbu.edu.tr

**Keywords:** endometrial cancer, sentinel lymph node mapping, minimally invasive surgery, surgical staging, robotic surgery, vNOTES

## Abstract

**Background**: Sentinel lymph node (SLN) mapping has become an established alternative to systematic lymphadenectomy for surgical staging in endometrial cancer. This study evaluated SLN mapping rates, anatomical distribution, factors associated with mapping failure, and perioperative outcomes in patients with early-stage endometrial cancer. **Methods**: This retrospective cohort study included patients with early-stage endometrial cancer who underwent SLN mapping during minimally invasive surgical staging between September 2022 and January 2025. Overall and bilateral SLN detection rates were calculated. Anatomical distribution of SLNs, factors associated with mapping failure (including a hemipelvis-level generalized estimating equation [GEE] analysis), and perioperative outcomes according to surgical approach were analyzed. **Results**: A total of 165 patients were included. The overall SLN detection rate was 96.4%, and bilateral mapping was achieved in 125 patients (75.8%). A total of 584 SLNs were removed, most commonly from the iliac basin. In the hemipelvis-level GEE analysis (330 hemipelves, 46 failures), ICG use (adjusted odds ratio [OR] 4.50, 95% confidence interval [CI] 1.07–18.83) and, in a sensitivity model, conventional laparoscopy compared with robotic surgery (OR 2.31, 95% CI 1.14–4.69) were associated with higher odds of mapping failure, whereas age and BMI were not. Operative time and intraoperative complication rates were comparable among laparoscopic, robotic-assisted, and vaginal natural orifice transluminal endoscopic surgery (vNOTES) procedures. Postoperative complication rates and length of hospital stay differed significantly across surgical approaches (both *p* = 0.002); post hoc pairwise comparison of complication rates showed that the difference was driven mainly by conventional laparoscopy compared with robotic-assisted surgery, whereas the small vNOTES group (n = 8) did not differ significantly from either approach and does not permit conclusions on its comparative safety. **Conclusions**: SLN mapping demonstrated high overall detection rates and acceptable bilateral mapping success in early-stage endometrial cancer. These findings support the feasibility of SLN mapping as a minimally invasive staging strategy in routine clinical practice.

## 1. Introduction

Endometrial cancer is the most common gynecologic malignancy in high-income countries. In 2022, an estimated 420,242 new cases and 97,704 deaths occurred worldwide [1]. According to the World Health Organization (WHO), 5463 women in Türkiye were diagnosed with endometrial cancer in 2018, with 1051 related deaths [2]. Over the past 30 years, the global incidence has increased by 132%, largely driven by rising obesity rates and increased life expectancy [3]. The primary prognostic factor in endometrial cancer is the stage at diagnosis. When detected at an early stage, the 5-year survival rate exceeds 90%; however, in disease with distant spread, it drops to approximately 22% [4,5]. In early-stage disease, lymph node involvement is a key determinant of prognosis and guides adjuvant treatment decisions [6,7]. Therefore, accurate lymph node assessment is essential for proper staging and treatment planning. Systematic lymphadenectomy has long been utilized in gynecologic oncology for nodal evaluation. However, it carries significant morbidity, including risks of lymphedema, lymphocele formation, and neurological complications [8,9,10,11]. The procedure is particularly challenging in obese patients, who represent a large portion of the endometrial cancer population. Additionally, given that most endometrial cancers are diagnosed at an early stage, the actual incidence of lymphatic metastasis is relatively low, making full lymphadenectomy unnecessary for many patients [12,13].

In this context, sentinel lymph node (SLN) mapping has gained prominence as a minimally invasive technique that enables accurate assessment of nodal status while reducing surgical morbidity. Several prospective studies have demonstrated high sensitivity and negative predictive value with SLN mapping, even in high-risk patients [12,13]. The FIRES trial reported a sensitivity of 97.2% and a negative predictive value of 99.6% in clinical stage I endometrial cancer patients undergoing robotic surgery [13]. Similarly, the SENTOR study by Cusimano et al. showed a sensitivity of 96% and a negative predictive value of 99% in intermediate- and high-grade tumors [14]. A meta-analysis by Marchocki et al., encompassing 429 patients across nine prospective studies, reported a pooled sensitivity of 92%, a false-negative rate of 8%, and a negative predictive value of 97% in high-grade endometrial cancers [15].

Beyond its diagnostic performance, recent large-scale meta-analyses have confirmed that SLN mapping offers comparable overall and progression-free survival outcomes to systematic lymphadenectomy. A meta-analysis including over 36,000 patients further supported these findings, revealing no significant differences in survival outcomes between SLN mapping and lymphadenectomy, and even suggesting improved overall survival in intermediate- to high-risk subgroups [16]. Furthermore, SLN mapping is associated with reduced intraoperative blood loss, minimizes the risk of surgical injuries, and lowers the incidence of complications such as chronic lymphedema and nerve damage, thereby reinforcing its clinical value in surgical staging [10,17].

Despite its advantages, SLN mapping is not without limitations. Previous studies have demonstrated that the success rates of bilateral SLN mapping in endometrial cancer vary widely, ranging from 52% to 96%, depending on factors such as surgical technique, tracer type, injection site, and patient characteristics [11,13]. A retrospective cohort study of 819 patients undergoing minimally invasive staging identified increasing body mass index (BMI) and high-risk histologic subtypes as independent predictors of mapping failure. Additionally, patients with macrometastases or micrometastases were more likely to experience unsuccessful mapping compared to those with negative SLNs or isolated tumor cells (ITCs) [18]. 

The aim of this study is to evaluate the clinical characteristics and surgical outcomes of patients with endometrial cancer who underwent laparoscopic, robotic-assisted, or vaginal natural orifice transluminal endoscopic surgery (vNOTES) staging with sentinel lymph node (SLN) mapping in our department, with particular emphasis on SLN detection rates and factors associated with mapping failure, and to share our institutional experience in the context of current evidence.

## 2. Materials and Method

The study was initiated with the approval of the hospital’s ethics committee and was conducted in accordance with the Declaration of Helsinki (approval number: AEŞH-BADEK-2024-1214). 

In this retrospective cohort study, patients who underwent laparoscopic or robotic staging surgery for endometrial cancer at the Gynecologic Oncology Department of our hospital between 1 September 2022 and 1 January 2025 were evaluated. Medical records of 201 patients operated on during this period were reviewed retrospectively. Demographic characteristics, preoperative data, surgical details, postoperative staging results, treatment plans, and follow-up findings were obtained from the electronic medical record system and analyzed.

Inclusion criteria were defined as having a final pathology report confirming a diagnosis of endometrial cancer, having undergone surgery at our hospital’s Gynecologic Oncology Department, and having received minimally invasive (laparoscopic, robotic-assisted, or vNOTES) staging surgery with SLN mapping. Exclusion criteria included a final pathology diagnosis other than endometrial cancer, staging surgery performed via laparotomy, and patients who underwent surgery at an external center.

Staging was performed according to the 2023 criteria of the International Federation of Gynecology and Obstetrics (FIGO). For patients treated prior to 2023, surgical and pathological findings were retrospectively adapted to align with the FIGO 2023 staging system. As part of the preoperative evaluation, tumor markers such as cancer antigen 125 (CA 125), carbohydrate antigen 19-9 (CA 19-9), cancer antigen 15-3 (CA 15-3), and alpha-fetoprotein (AFP) were assessed, and thoracic, abdominal, and pelvic computed tomography scans were performed to rule out distant disease.

All procedures were conducted by the same experienced onco-gynecologic surgical team, proficient in advanced endoscopic surgery, using conventional laparoscopy, robotic-assisted techniques, or the vaginal natural orifice transluminal endoscopic surgery (vNOTES) approach. For SLN mapping, methylene blue and indocyanine green (ICG; 2.5 mg/mL, Pulsion Medical Systems, Feldkirchen, Germany) were used as tracers. Methylene blue was used undiluted, whereas ICG was diluted 1:1 with 0.9% normal saline. A total of 4 mL of the prepared dye solution was injected into the cervix at the 3 and 9 o’clock positions; at each site, 1 mL was administered deeply (approximately 1 cm) and 1 mL superficially (approximately 5 mm) using a 22-gauge spinal needle. Each injection was delivered over approximately 10 s. Dye injections were performed at the beginning of the procedure, following carbon dioxide insufflation, and prior to robotic system docking.

SLNs were identified using either the PINPOINT Endoscopic Fluorescence Imaging System (ICG Mode, Near-Infrared Fluorescence Mode, Stryker, Kalamazoo, MI, USA) or the Da Vinci Xi robotic surgical system (Intuitive Surgical Inc., Sunnyvale, CA, USA) in cases where methylene blue and ICG were utilized.

All cases were managed in accordance with the steps outlined in the SLN algorithm. Briefly, following inspection of the peritoneal cavity and evaluation of the pelvic and abdominal structures, the retroperitoneal space was accessed. Anatomical spaces, including the pararectal, paravesical, and obturator regions, were systematically created. SLN mapping was then visualized. In addition to the mapped SLNs, any suspicious bulky lymph nodes outside the mapping areas were also excised. 

Successful SLN mapping was defined as identification of bilateral SLNs, whereas failed SLN mapping was defined as unilateral or no SLN mapping. In cases of failed mapping, the surgeons performed side-specific full lymphadenectomy regardless of intraoperative tumor characteristics. The overall detection rate was defined as successful identification of at least one SLN, regardless of whether mapping was unilateral or bilateral. For anatomical classification, the common iliac bifurcation region was defined as the anatomical region extending from the level at which the common iliac artery bifurcates into the external and internal iliac arteries and encompassing the space between these two vessels.

Operative time was defined as the time from the first skin incision to the last skin closure in the laparoscopic and vNOTES groups. In robotic-assisted procedures, SLN mapping was performed before robot docking, and operative time was recorded from the completion of docking to the last skin closure; the time required for SLN mapping before docking is therefore not included in the robotic group. Intraoperative complications were defined as any event occurring during the surgical procedure, while postoperative complications included both early (≤30 days) and late (31–90 days) events. 

For analyses evaluating factors associated with bilateral SLN mapping success, BMI was categorized according to the WHO classification as normal weight (18.5–24.9 kg/m^2^), overweight (25.0–29.9 kg/m^2^), obesity class I (30.0–34.9 kg/m^2^), obesity class II (35.0–39.9 kg/m^2^), and obesity class III (≥40.0 kg/m^2^). 

SLNs were routinely serially sectioned at 100–200 µm intervals for detailed microscopic evaluation. All sections were initially stained with hematoxylin and eosin (H&E) as part of a standardized ultrastaging protocol. SLNs that were negative on routine H&E examination subsequently underwent cytokeratin immunohistochemical staining (PanCK/AE1-AE3) to facilitate the detection of occult tumor deposits, including ITCs and micrometastases. Non-sentinel lymph nodes were evaluated using conventional H&E staining only. Tumor deposits measuring > 2 mm were classified as macrometastases, those measuring 0.2–2 mm as micrometastases, and deposits < 0.2 mm as ITCs.

### 2.1. Statistical Analysis

All statistical analyses were performed using SPSS software (IBM SPSS Statistics for Windows, Version 25.0; IBM Corp., Armonk, NY, USA). Continuous variables were assessed for normality using the Shapiro–Wilk test and presented as mean ± standard deviation (SD) or median with interquartile range (IQR), as appropriate. Categorical variables were expressed as frequencies and percentages. Group comparisons were performed using Pearson’s chi-square test or Fisher’s exact test for categorical variables and Student’s *t*-test or Mann–Whitney U test for continuous variables, depending on data distribution.

SLN mapping success was evaluated separately for each hemipelvis, along with bilateral mapping success and overall detection rates. To account for the limitations of patient-level analyses combining unilateral and bilateral mapping failures, an additional hemipelvis-based analysis was conducted using generalized estimating equations (GEE) with a binomial distribution and logit link function. Each hemipelvis was treated as a separate observation, with within-patient correlation accounted for using an exchangeable working correlation structure and robust standard errors. Mapping failure was defined as unsuccessful SLN mapping on the corresponding hemipelvis. The adjusted model included age, BMI, and tracer type. A sensitivity analysis additionally adjusting for surgical approach was performed in the subgroup undergoing laparoscopic or robotic surgery. Odds ratios (ORs) with 95% confidence intervals (CIs) for mapping failure are reported, with methylene blue and robotic-assisted surgery as the reference categories.

All tests were two-sided, and *p* < 0.05 was considered statistically significant.

### 2.2. Use of Generative AI

Generative artificial intelligence (AI) tools (OpenAI ChatGPT-5.5) were used exclusively for English language editing and grammatical refinement of the manuscript, as well as well as to assist in the initial preparation of a schematic illustration included in the manuscript. The illustration was subsequently reviewed, modified, annotated, and finalized by the authors using the original study data. The AI tools were not used for study design, data collection, statistical analysis, data interpretation, or scientific decision-making. All scientific content was independently verified and approved by the authors.

## 3. Results

### 3.1. Patient Characteristics and Surgical Approach

**Figure 1:**
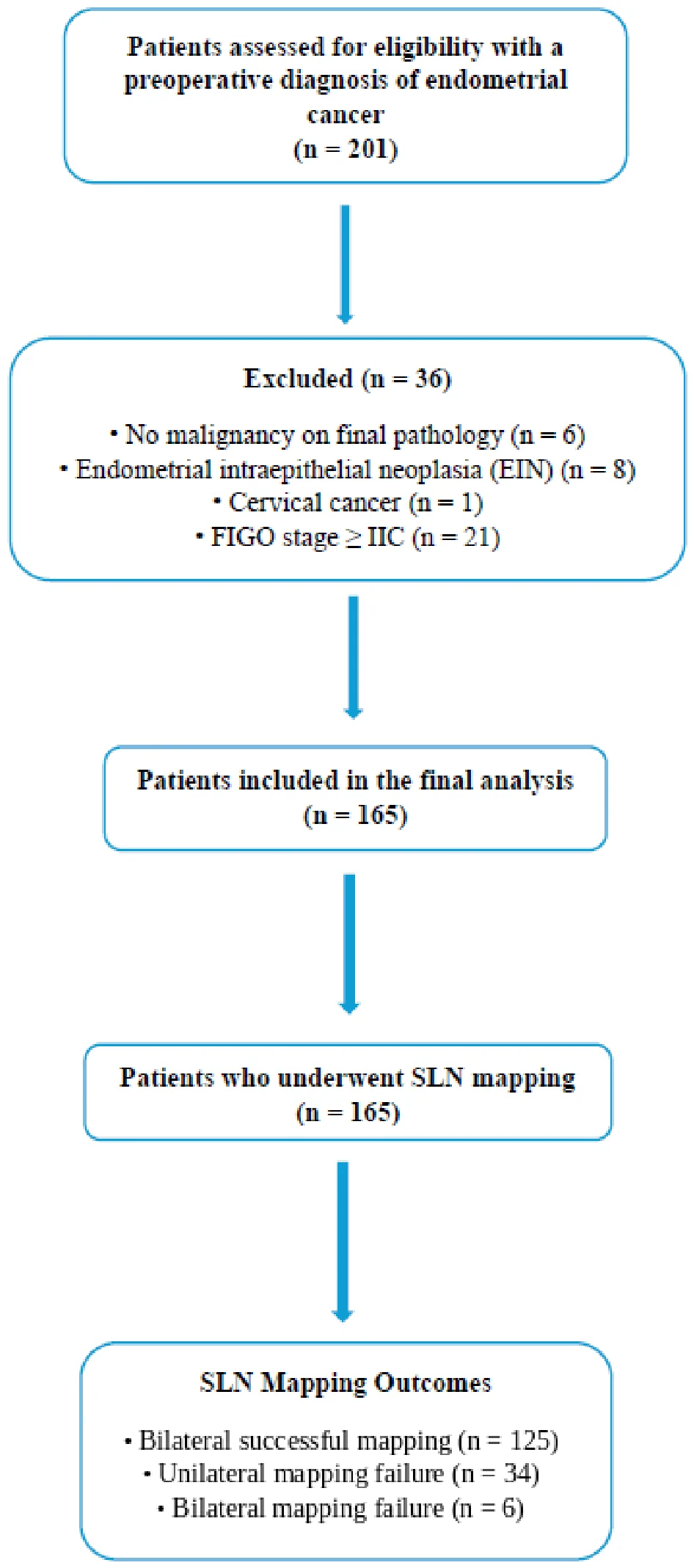
Flowchart of patient selection and sentinel lymph node mapping outcomes.

A total of 201 patients with a preoperative diagnosis of endometrial cancer were initially evaluated for inclusion in the study. After applying the predefined exclusion criteria, 36 patients were excluded due to absence of malignancy on final pathology (n = 6), a diagnosis of endometrial intraepithelial neoplasia (EIN) (n = 8), cervical cancer (n = 1), or advanced-stage disease (FIGO stage IIC or higher) (n = 21). Consequently, 165 patients with early-stage endometrial cancer were included in the final analysis (Figure 1).

Regarding surgical history, 115 patients (69.7%) had no previous abdominal or non-cesarean pelvic surgery, whereas 39 (23.6%) had a history of abdominal surgery only, 9 (5.5%) had undergone non-cesarean pelvic surgery only, and 2 (1.2%) had a history of both abdominal and non-cesarean pelvic procedures. Previous cesarean delivery, which was analyzed separately and may overlap with these categories, was recorded in 39 patients (23.6%) (Table 1).

All patients underwent minimally invasive surgery. Robotic-assisted surgery was the most frequently utilized approach, accounting for 109 cases (66.1%), followed by conventional laparoscopy in 48 patients (29.1%) and vNOTES in 8 patients (4.8%) (Table 1).

### 3.2. Sentinel Lymph Node Mapping Outcomes

SLN mapping was successfully achieved bilaterally in 125 of 165 patients (75.8%). Unilateral mapping failure occurred in 34 patients (20.6%), including 18 right-sided (10.9%) and 16 left-sided (9.7%) failures. Bilateral mapping failure was observed in six patients (3.6%). Overall, successful SLN detection on at least one pelvic side was achieved in 159 patients, corresponding to an overall detection rate of 96.4% (Table 2).

No macrometastases or micrometastases were identified in the SLNs. Two cases of ITCs were detected through ultrastaging. No metastatic non-sentinel lymph nodes were identified, including in patients who underwent side-specific lymphadenectomy following SLN mapping failure. 

### 3.3. Anatomical Distribution of Sentinel Lymph Nodes

**Figure 2:**
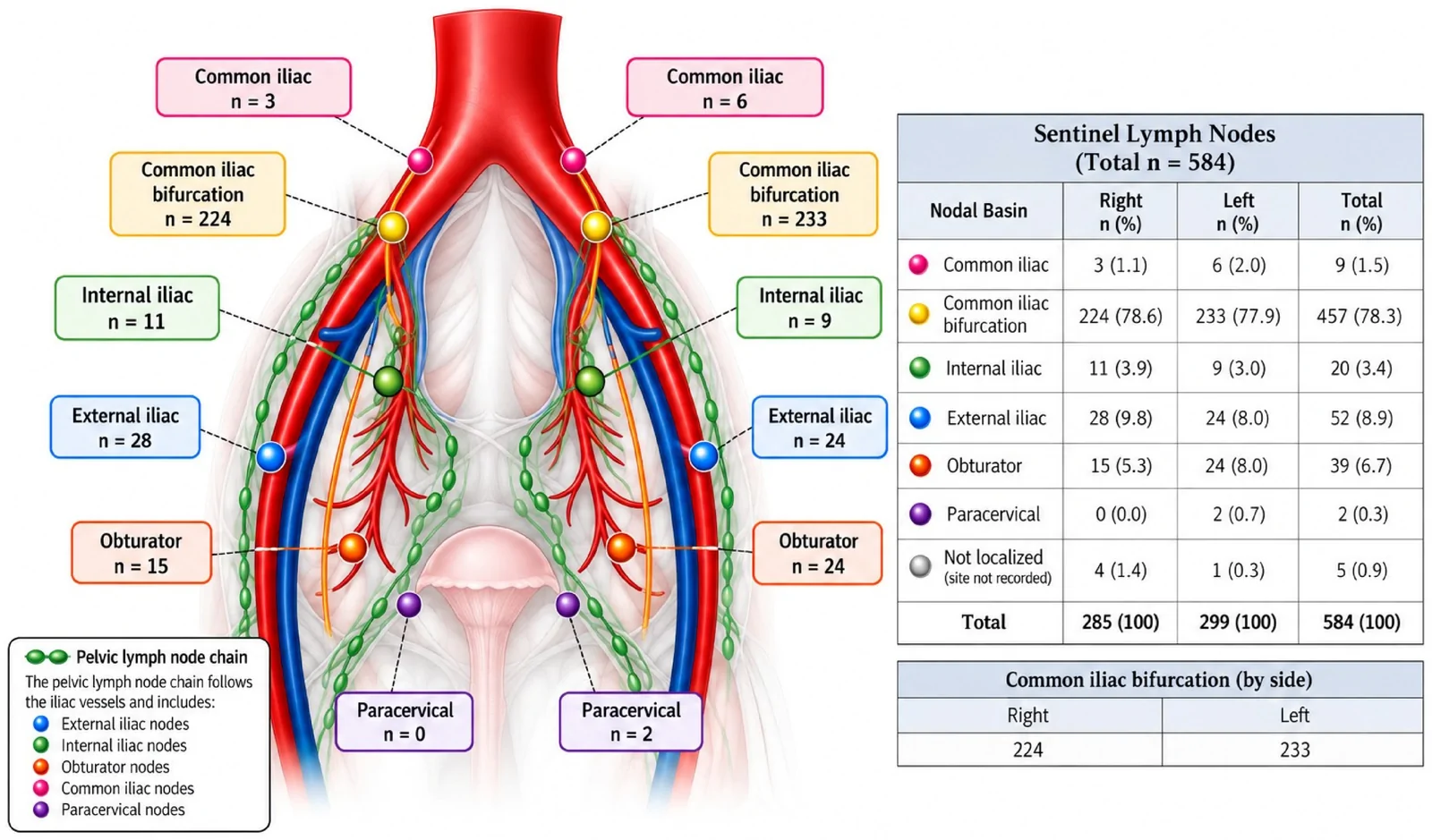
**Anatomical distribution of sentinel lymph nodes identified during sentinel lymph node mapping in patients with early-stage endometrial cancer**. Numbers indicate the total number of sentinel lymph nodes detected within each anatomical nodal basin. A total of 584 sentinel lymph nodes were retrieved, including 285 from the right hemipelvis and 299 from the left hemipelvis.

A total of 584 SLNs were retrieved during the study period, including 285 from the right hemipelvis and 299 from the left hemipelvis. The median number of SLNs removed per patient was 2 (range, 1–10). The common iliac bifurcation was the most frequent anatomical location, accounting for 224 (78.6%) and 233 (77.9%) of the retrieved SLNs in the right and left hemipelvis, respectively. Overall, 457 SLNs (78.3%) were identified in this region. The external iliac region was the second most frequent location (n = 52, 8.9%), followed by the obturator (n = 39, 6.7%) and internal iliac regions (n = 20, 3.4%). SLNs were less frequently identified in the common iliac (n = 9, 1.5%) and paracervical regions (n = 2, 0.3%). The anatomical locations of five retrieved SLNs (0.9%), including four from the right hemipelvis and one from the left hemipelvis, were not recorded and are presented separately in Table 3 The anatomical distribution of retrieved SLNs is illustrated in Figure 2. Patient-level mapping outcomes are reported separately in Table 2.

### 3.4. Factors Associated with Sentinel Lymph Node Mapping Failure

Factors associated with SLN mapping failure were evaluated by comparing patients with successful bilateral mapping (n = 125) and those with mapping failure (n = 40). No significant differences were observed between the groups with respect to age, menopausal status, history of vaginal delivery, previous cesarean section, BMI category, uterine volume, number of comorbidities, surgical approach, LVSI status, depth of myometrial invasion, or tumor grade (all *p* > 0.05) (Table 4).

Although statistical significance was not reached, borderline differences were observed for previous non-cesarean pelvic surgery (*p* = 0.067) and tracer type (*p* = 0.075; Fisher’s exact *p* = 0.107). Previous non-cesarean pelvic surgery was observed only among patients with successful bilateral SLN mapping. Although the distribution of tracer type differed between the successful and failed mapping groups, no statistically significant association with bilateral SLN mapping success was identified.

At the patient level, no patient-, tumor-, or surgery-related factor reached statistical significance for SLN mapping failure in the present cohort.

Hemipelvis-level analysis. A total of 330 hemipelves from 165 patients were analyzed, and mapping failed in 46 (13.9%). Failure occurred in 44 of 286 hemipelves (15.4%) mapped with ICG and in 2 of 44 (4.5%) mapped with methylene blue. In the unadjusted generalized estimating equation (GEE) model, ICG was associated with higher odds of failure than methylene blue (odds ratio [OR] 3.82, 95% confidence interval [CI] 0.92–15.90; *p* = 0.066). After adjustment for age and BMI, the association was statistically significant (OR 4.50, 95% CI 1.07–18.83; *p* = 0.040), whereas age (OR 1.00, 95% CI 0.96–1.05; *p* = 0.891) and BMI (OR 1.03, 95% CI 0.98–1.09; *p* = 0.258) were not associated with failure. In the sensitivity analysis restricted to laparoscopic and robotic procedures (156 patients, 312 hemipelves), ICG (OR 4.80, 95% CI 1.05–22.02; *p* = 0.043) and conventional laparoscopy compared with robotic surgery (OR 2.31, 95% CI 1.14–4.69; *p* = 0.021) were associated with higher odds of failure, whereas age and BMI remained non-significant. Failure occurred in 19 of 94 laparoscopic hemipelves (20.2%) and 27 of 218 robotic hemipelves (12.4%), and in none of the 16 vNOTES hemipelves.

### 3.5. Surgical Outcomes According to Operative Approach

Surgical outcomes according to operative approach are summarized in Table 5. The median operative time was comparable among laparoscopic, robotic-assisted, and vNOTES procedures (195, 190, and 180 min, respectively; *p* = 0.833). The shortest operative time (24 min) was recorded in the robotic-assisted group, in an elderly, thin patient with favorable anatomy (small uterus, easy access, and no adhesions). In this case, SLN mapping was performed before robot docking, and the recorded operative time started after docking. Conversion to laparotomy was required in only one patient undergoing conventional laparoscopy, whereas no conversions occurred in the robotic-assisted or vNOTES groups (*p* = 0.339).

Intraoperative complication rates did not differ significantly among the three surgical approaches (*p* = 0.428). Overall postoperative complication rates and length of hospital stay differed significantly across surgical approaches (*p* = 0.002 for both comparisons; Table 5). In a post hoc, unadjusted pairwise comparison of postoperative complication rates (Fisher’s exact test), the difference was mainly attributable to conventional laparoscopy versus robotic-assisted surgery (16/48, 33.3% vs. 13/109, 11.9%; *p* = 0.003), whereas the vNOTES group (0/8) did not differ significantly from laparoscopy (*p* = 0.089) or robotic-assisted surgery (*p* = 0.595). However, these findings should be interpreted cautiously, particularly for the vNOTES group (n = 8), as patients were selected based on their suitability for this approach and the small sample size precludes reliable conclusions regarding its comparative safety.

No significant differences were observed among the groups regarding the administration of adjuvant treatment or completion surgery (*p* = 0.105).

## 4. Discussion

In this retrospective single-center study, SLN mapping achieved an overall detection rate of 96.4% and a bilateral mapping rate of 75.8% in patients with early-stage endometrial cancer. The iliac region was the most common anatomical site for SLN detection. In addition, age and BMI were not associated with mapping failure, whereas ICG use and conventional laparoscopy were associated with higher odds of hemipelvis-level failure in exploratory GEE analyses. Although statistically significant differences were observed in postoperative complication rates and length of hospital stay among the minimally invasive surgical approaches, these findings should be interpreted with caution because of the limited number of patients in the vNOTES group. Post hoc pairwise testing indicated that the higher postoperative complication rate was mainly associated with conventional laparoscopy (33.3%) compared with robotic-assisted surgery (11.9%), while no vNOTES patient had a complication; this difference should not be attributed to the vNOTES approach. Overall, our findings support the feasibility and high detection rates of incorporating SLN mapping into minimally invasive surgical staging for early-stage endometrial cancer and provide real-world evidence regarding the implementation of a standardized SLN mapping algorithm in routine clinical practice.

The high overall detection rate observed in our cohort aligns with the increasing evidence supporting the reliability of SLN mapping in endometrial cancer. Large prospective studies have documented overall detection rates between approximately 86% and 97%, with bilateral mapping rates varying significantly based on surgical expertise, tracer selection, and adherence to mapping algorithms. In this study, the overall detection rate of 96.4% and bilateral mapping rate of 75.8% are within the upper range of values reported in the literature. These findings indicate that SLN mapping can be effectively implemented in routine clinical practice within a tertiary referral center, achieving outcomes comparable to those reported by high-volume institutions [13,14,15].

The anatomical distribution of SLNs observed in our cohort was consistent with previous reports. The common iliac bifurcation was the most common site of SLN detection, followed by the external iliac, obturator, and internal iliac regions, whereas common iliac and paracervical nodes were encountered only rarely. Similar findings have been described in earlier studies, which demonstrated that SLNs are predominantly located within the pelvic nodal basins, particularly along the iliac and obturator pathways [13,14,19]. The concordance between our findings and the existing literature further supports the reliability of cervical tracer injection for mapping the primary lymphatic drainage pathways of the uterus [13,14].

Another important finding of the present study was the absence of a significant patient-level association between mapping failure and most patient- and tumor-related characteristics, including age and BMI. Variables that have previously been suggested to influence mapping performance, including age, BMI, surgical approach, prior abdominal or pelvic surgery, uterine size, tumor grade, and depth of myometrial invasion, were not associated with bilateral mapping success in the patient-level analysis [13,14,19,20]. In the hemipelvis-level GEE analysis, however, ICG use and conventional laparoscopy were associated with higher odds of mapping failure (see below). Previous pelvic surgery and tracer type also demonstrated borderline statistical significance at the patient level, although neither reached the predefined threshold for significance. The lack of association with age and BMI suggests that SLN mapping can be successfully implemented across a broad spectrum of patients with early-stage endometrial cancer and may be less dependent on individual patient characteristics than previously assumed [21]. Nevertheless, the relatively limited number of mapping failures in our cohort may have reduced the statistical power to detect small differences between groups.

Because obesity is a well-recognized risk factor for endometrial cancer and may increase the technical complexity of SLN mapping, we further evaluated mapping outcomes according to clinically relevant BMI categories, including class III obesity. In our cohort, BMI category was not significantly associated with successful bilateral SLN mapping. This finding suggests that high BMI alone may not adversely affect SLN detection when contemporary minimally invasive surgical techniques are used. Recent evidence has suggested that robotic surgery may help overcome obesity-related technical challenges by improving visualization and instrument dexterity, thereby maintaining high SLN detection rates even in patients with severe obesity. Although our study was not designed to evaluate the interaction between BMI and surgical approach, our findings are consistent with the notion that obesity should not preclude the use of SLN mapping in appropriately selected patients [22].

The comparison of minimally invasive surgical approaches revealed similar operative times and intraoperative complication rates across laparoscopic, robotic-assisted, and vNOTES procedures. Although statistically significant differences were observed in postoperative complication rates and length of hospital stay among the three surgical approaches, these findings should be interpreted with caution because only a small number of patients underwent vNOTES (n = 8). Previous studies have demonstrated that minimally invasive surgical techniques are associated with reduced perioperative morbidity and enhanced postoperative recovery in patients with endometrial cancer [7,23,24]. However, given the retrospective design of our study and the limited size of the vNOTES subgroup, further prospective studies are required to determine whether the observed differences are attributable to the surgical approach itself or to patient selection and institutional practice patterns.

Two variables, previous non-cesarean pelvic surgery and tracer type, showed borderline associations with bilateral mapping success at the patient level (*p* = 0.067 and *p* = 0.075, respectively), and the direction of these associations deserves comment. Previous pelvic surgery is generally expected to impair mapping, because postoperative adhesions, scarring and interrupted lymphatic channels may obstruct or divert tracer migration from the cervix to the pelvic nodes. In our cohort, however, no mapping failure occurred among patients with previous pelvic surgery (Table 3), which is opposite to this hypothesis. As no plausible protective mechanism exists, and considering the very small size of this subgroup and the absence of adjustment for multiple comparisons, this finding is most likely attributable to chance. This interpretation is consistent with the available evidence: a meta-analysis found no significant association between failed mapping and prior pelvic surgery (OR 0.86, *p* = 0.55), prior cesarean section or lysis of adhesions [25], and intraoperative adhesiolysis was not an independent predictor of failed mapping in two multicenter series using intracervical ICG [26,27]. The tracer finding was also contrary to expectation. In the hemipelvis-level GEE analysis, mapping failed more often with ICG than with methylene blue (15.4% versus 4.5% of hemipelves; adjusted OR 4.50, 95% CI 1.07–18.83; *p* = 0.040), and the association persisted after additional adjustment for surgical approach (OR 4.80, 95% CI 1.05–22.02; *p* = 0.043), whereas randomized and pooled data indicate that ICG with near-infrared imaging detects substantially more sentinel nodes than blue dyes (97% versus 47% of nodes compared with isosulfan blue in the FILM trial) [28,29]. This advantage has been attributed to the properties of ICG: about 98% of the dye is bound to plasma proteins, mainly albumin, and its fluorescence peaks at approximately 830 nm within the near-infrared window, which avoids tissue autofluorescence and permits detection through up to 10–15 mm of tissue, whereas blue dyes must be visualized directly [30]. These properties would be expected to favor, rather than hinder, bilateral detection with ICG, so that a true biological disadvantage of ICG seems unlikely. The estimate is imprecise, because only 22 patients (44 hemipelves) were mapped with methylene blue and only two of these hemipelves failed. Tracers were allocated retrospectively and non-randomly, so residual confounding by surgeon, period, imaging system or case selection cannot be excluded, and no adjustment was made for multiple comparisons. Although the injection volume was standardized in our protocol (4 mL in total), whereas a cervical ICG dose below 3 mL has been associated with failed mapping in a meta-analysis [25], other technical aspects of ICG preparation and injection could not be evaluated. Conventional laparoscopy was also associated with higher odds of failure than robotic-assisted surgery (OR 2.31, 95% CI 1.14–4.69; *p* = 0.021). Possible explanations include differences between the imaging systems used (PINPOINT and Da Vinci Xi), in visualization, and in the learning curve over the study period, but these explanations are hypotheses that cannot be tested in this retrospective dataset. Both findings should therefore be regarded as exploratory and hypothesis-generating, and larger prospective studies with predefined tracer allocation and standardized imaging are needed to clarify them.

The present study has several strengths. It represents one of the largest single-center experiences evaluating SLN mapping in endometrial cancer from Türkiye and reflects the real-world implementation of a standardized SLN mapping algorithm in a tertiary referral center. In addition, all procedures were performed using minimally invasive techniques, allowing assessment of mapping outcomes across different contemporary surgical approaches. Furthermore, the detailed evaluation of the anatomical distribution of SLNs provides additional information regarding lymphatic drainage patterns in patients with early-stage endometrial cancer. Although the feasibility of SLN mapping has been well established in prospective trials, real-world data reflecting the implementation of standardized SLN mapping algorithms in routine clinical practice remain valuable, particularly from regions where published institutional experience is limited.

Nevertheless, several limitations should be acknowledged. First, the retrospective design introduces an inherent risk of selection and information bias. Second, this was a single-center study, which may limit the generalizability of the findings. Third, although factors associated with bilateral SLN mapping failure were evaluated, multivariable logistic regression analysis was not performed because the number of mapping failure events (n = 40) was insufficient to support a reliable multivariable model without increasing the risk of overfitting. Therefore, the identified associations should be interpreted with caution. Fourth, the relatively small number of patients in the vNOTES group may have limited the ability to perform robust comparisons among surgical approaches. Likewise, the numbers of patients with previous pelvic surgery and of those in whom methylene blue was used were small, so the borderline associations observed for these variables should be considered exploratory. Fifth, because failure was defined as unilateral or absent mapping, pooling one-sided and two-sided failures may mask side-specific differences; the hemipelvis-level GEE analysis addressed this only partially, because only 22 patients received methylene blue (two failed hemipelves), and its estimates were imprecise and not adjusted for multiplicity. Sixth, because completion lymphadenectomy was performed only side-specifically after failed mapping, sensitivity, negative predictive value, and the false-negative rate could not be assessed; the study therefore describes mapping rates and feasibility rather than diagnostic accuracy. Seventh, operative time in the robotic-assisted group was recorded from docking, whereas SLN mapping was performed before docking; robotic operative times therefore do not include the SLN phase and may underestimate total procedure time compared with the laparoscopic and vNOTES groups, in which time was recorded from skin incision. Finally, long-term oncologic outcomes, including recurrence and survival, were not evaluated, precluding assessment of the impact of SLN mapping on long-term clinical outcomes.

## 5. Conclusions

SLN mapping demonstrated a high overall detection rate and an acceptable bilateral mapping rate in patients with early-stage endometrial cancer undergoing minimally invasive surgical staging. The majority of SLNs were identified within the iliac nodal basins, consistent with established lymphatic drainage patterns. Age and BMI were not associated with mapping failure, whereas ICG use and conventional laparoscopy were associated with higher failure odds in exploratory hemipelvis-level analyses. Although differences in postoperative morbidity and length of hospital stay were observed among the surgical approaches, these findings should be interpreted with caution because of the limited number of patients who underwent vNOTES. Overall, these findings support the feasibility and high detection rates of SLN mapping as a minimally invasive staging strategy in routine clinical practice. Further prospective multicenter studies are warranted to validate these findings and evaluate long-term oncologic outcomes.

## Figures and Tables

**Table 1:** Baseline Surgical Characteristics of the Study Population.

Characteristic	n (%)
**Previous surgical history**	
No previous abdominal or non-cesarean pelvic surgery	115 (69.7)
Previous abdominal surgery only	39 (23.6)
Previous non-cesarean pelvic surgery only	9 (5.5)
Previous abdominal and non-cesarean pelvic surgery	2 (1.2)
Previous cesarean delivery *	39 (23.6)
**Surgical approach**	
Laparoscopic surgery	48 (29.1)
Robotic-assisted surgery	109 (66.1)
vNOTES	8 (4.8)

* Cesarean delivery is reported separately and may overlap with the surgical history categories. Bold text indicates category headings used to organize the variables in the table.

**Table 2:** Distribution of Sentinel Lymph Node Mapping Outcomes Among 165 Patients.

Mapping Outcome	Number of Patients (n)	Percentage (%)
Successful bilateral mapping	125	75.8%
Unilateral mapping failure	34	20.60%
Right side failure	18	10.90%
Left side failure	16	9.69%
Bilateral mapping failure	6	3.63%
**Total**	**165**	**100%**

Bold formatting is used to distinguish the table headers and the total row.

**Table 3:** Anatomical Distribution of Retrieved Sentinel Lymph Nodes.

SLN Location	Right Hemipelvis (Nodes)	Left Hemipelvis (Nodes)	Total (n)
Obturator	15	24	39
External iliac	28	24	52
Internal iliac	11	9	20
Common iliac	3	6	9
Common iliac Bifurcation	224	233	457
Paracercival	0	2	2
Not localized (site not recorded)	4	1	5
**Total**	**285**	**299**	**584**

SLN, sentinel lymph node; n, number of lymph nodes. ‘Not localized (site not recorded)’ refers to retrieved sentinel lymph nodes for which the anatomical location was not documented. Bold formatting indicates the total row.

**Table 4:** Comparison of Clinical and Surgical Characteristics According to Sentinel Lymph Node Mapping Success.

Clinical Variable	Total n = 165	Successful SLN Mapping n = 125	Failed SLN Mapping n = 40	*p* Value
Age at diagnosis, mean ± SD (years)	58.00 ± 9.97	57.98 ± 9.77	58.05 ± 10.72	0.968
Menopausal status				0.891
Yes	121 (73.3)	92 (73.6)	29 (72.5)	
No	44 (26.7)	33 (26.4)	11 (27.5)	
Previous deliveries				0.760
Yes	150 (90.9)	114 (91.2)	36 (90.0)	
No	15 (9.1)	11 (8.8)	4 (10.0)	
Previous cesarean deliveries				0.509
Yes	39 (23.6)	28 (22.4)	11 (27.5)	
No	126 (76.4)	97 (77.6)	29 (72.5)	
Previous pelvic surgery (non-cesarean)				0.067 *
Yes	11 (6.7)	11 (8.8)	0 (0.0)	
No	154 (93.3)	114 (91.2)	40 (100.0)	
Previous abdominal surgery				0.198
Yes	41 (24.8)	28 (22.4)	13 (32.5)	
No	124 (75.2)	97 (77.6)	27 (67.5)	
BMI at diagnosis, median (range), kg/m^2^	32 (21–60)	32 (21–59)	31.5 (23–60)	0.604
BMI category (WHO)				0.321 *
Normal	15 (9.1)	14 (11.2)	1 (2.5)	
Overweight	32 (19.4)	22 (17.6)	10 (25.0)	
Obesity Class I	55 (33.3)	42 (33.6)	13 (32.5)	
Obesity Class II	35 (21.2)	28 (22.4)	7 (17.5)	
Obesity Class III	28 (17.0)	19 (15.2)	9 (22.5)	
Number of comorbidities, median (range)	1 (0–5)	1 (0–5)	1 (0–3)	0.086
Uterine volume (cm^3^), median (range)	112.316 (2.68–402.19)	108.927 (2.68–402.19)	114.243 (38–348.33)	0.936
Type of surgery				0.078 *
Laparoscopy (including VAH + LS)	48 (29.1)	32 (25.6)	16 (40.0)	
Robotic-assisted	109 (66.1)	85 (68.0)	24 (60.0)	
vNOTES	8 (4.8)	8 (6.4)	0 (0.0)	
Type of tracer				0.075 †
Indocyanine green (ICG)	143 (86.7)	105 (84.0)	38 (95.0)	
Methylene blue	22 (13.3)	20 (16.0)	2 (5.0)	
Lymphovascular space invasion (LVSI) ‡				0.448 *
No LVSI	147 (89.1)	113 (90.4)	34 (85.0)	
Focal LVSI	10 (6.1)	6 (4.8)	4 (10.0)	
Substantial LVSI	8 (4.8)	6 (4.8)	2 (5.0)	
Myometrial invasion				0.936 *
No invasion	41 (24.8)	32 (25.6)	9 (22.5)	
<50% invasion	97 (58.8)	73 (58.4)	24 (60.0)	
≥50% invasion	27 (16.4)	20 (16.0)	7 (17.5)	
Tumor grading				0.140 *
Grade 1	142 (86.1)	111 (88.8)	31 (77.5)	
Grade 2	19 (11.5)	11 (8.8)	8 (20.0)	
Grade 3	4 (2.4)	3 (2.4)	1 (2.5)	

Data are presented as n (%) unless otherwise indicated; percentages are calculated within columns. Continuous variables are presented as mean ± SD (age) or median (range) and were compared using the independent-samples t-test or the Mann–Whitney U test, as appropriate. Categorical variables were compared using Pearson’s chi-square test or Fisher’s exact test (Fisher–Freeman–Halton exact test for variables with more than two categories). * Exact test (Fisher’s exact or Fisher–Freeman–Halton), used because of sparse cells. † Pearson chi-square test (Fisher’s exact *p* = 0.107). ‡ Fisher–Freeman–Halton exact test. Abbreviations: SLN, sentinel lymph node; WHO, World Health Organization; BMI, body mass index; SD, standard deviation; ICG, indocyanine green; LVSI, lymphovascular space invasion; vNOTES, vaginal natural orifice transluminal endoscopic surgery; VAH, vaginal-assisted hysterectomy; LS, laparoscopy.

**Table 5:** Comparison of Perioperative Outcomes Among Different Minimally Invasive Surgical Approaches.

Characteristic	Laparoscopy (n = 48)	Robotic Surgery (n = 109)	V-Notes (n = 8)	*p* Value
Operation time (min) Median Mean ± SD	195 (100–320) 194.06 ± 44.01	190 (24–340) 192.19 ± 51.37	180 (140–240) 185 ± 33.8	0.833
Conversion to laparotomy	1	0	0	0.339
Intra-operation complications No Yes	43 5	94 15	6 2	0.428
Post-operation complications None Clavien–Dindo grade II Clavien–Dindo grade IIIb	32 16 0	96 12 1	8 0 0	0.002 *
Length of hospital stays (days) Median (min-max)	3 (2–7)	2 (1–14)	2 (2–3)	**0.002**
Chemotherapy Radiotherapy Chemotherapy + radiotherapy Completion surgery No	2 7 2 2 35	0 19 1 2 87	0 0 1 0 7	0.105

* *p* value refers to the comparison of the overall postoperative complication rate among surgical approaches; complications are presented according to the Clavien–Dindo classification. Bold values indicate statistically significant results (*p* < 0.05).

## Data Availability

The data that support the findings of this study are available from the corresponding author upon reasonable request. The data are not publicly available due to privacy and ethical restrictions involving participant information.

## References

[1] Bray F., Laversanne M., Sung H., Ferlay J., Siegel R.L., Soerjomataram I., Jemal A. (2024). Global cancer statistics 2022: GLOBOCAN estimates of incidence and mortality worldwide for 36 cancers in 185 countries. CA Cancer J. Clin..

[2] Turkish Society of Gynecologic Oncology Uterine (Endometrial) Cancer. https://trsgo.org/files/toplum-icin/rahim-ici-endometrium-kanseri.pdf.

[3] Crosbie E.J., Kitson S.J., McAlpine J.N., Mukhopadhyay A., Powell M.E., Singh N. (2022). Endometrial cancer. Lancet.

[4] Trojano G., Olivieri C., Tinelli R., Damiani G.R., Pellegrino A., Cicinelli E. (2019). Conservative treatment in early-stage endometrial cancer: A review. Acta Biomed..

[5] American Cancer Society Survival Rates for Endometrial Cancer. https://www.cancer.org/cancer/endometrial-cancer/detection-diagnosis-staging/survival-rates.

[6] Amant F., Mirza M.R., Koskas M., Creutzberg C.L. (2018). Cancer of the corpus uteri. Int. J. Gynaecol. Obstet..

[7] Concin N., Matias-Guiu X., Vergote I., Cibula D., Mirza M.R., Marnitz S., Ledermann J., Bosse T., Chargari C., Fagotti A. (2021). ESGO/ESTRO/ESP guidelines for the management of patients with endometrial carcinoma. Int. J. Gynecol. Cancer.

[8] Benedetti Panici P., Basile S., Maneschi F., Lissoni A.A., Signorelli M., Scambia G., Angioli R., Tateo S., Mangili G., Katsaros D. (2008). Systematic pelvic lymphadenectomy vs. no lymphadenectomy in early-stage endometrial carcinoma: Randomized clinical trial. J. Natl. Cancer Inst..

[9] Biglia N., Librino A., Ottino M.C., Panuccio E., Daniele A., Chahin A. (2015). Lower limb lymphedema and neurological complications after lymphadenectomy for gynecological cancer. Int. J. Gynecol. Cancer.

[10] Leitao M.M., Zhou Q.C., Gomez-Hidalgo N.R., Iasonos A., Baser R., Mezzancello M., Chang K., Ward J., Chi D.S., Roche K.L. (2020). Patient-reported outcomes after surgery for endometrial carcinoma: Prevalence of lower-extremity lymphedema after sentinel lymph node mapping versus lymphadenectomy. Gynecol. Oncol..

[11] Geppert B., Lönnerfors C., Bollino M., Persson J. (2018). Sentinel lymph node biopsy in endometrial cancer: Feasibility, safety and lymphatic complications. Gynecol. Oncol..

[12] Persson J., Salehi S., Bollino M., Lönnerfors C., Falconer H., Geppert B. (2019). Pelvic sentinel lymph node detection in high-risk endometrial cancer (SHREC-trial): The final step towards a paradigm shift in surgical staging. Eur. J. Cancer.

[13] Rossi E.C., Kowalski L.D., Scalici J., Cantrell L., Schuler K., Hanna R.K., Method M., Ade M., Ivanova A., Boggess J.F. (2017). A comparison of sentinel lymph node biopsy to lymphadenectomy for endometrial cancer staging (FIRES trial): A multicentre, prospective, cohort study. Lancet Oncol..

[14] Cusimano M.C., Vicus D., Pulman K., Maganti M., Bernardini M.Q., Bouchard-Fortier G., Laframboise S., May T., Hogen L.F., Covens A.L. (2021). Assessment of sentinel lymph node biopsy vs lymphadenectomy for intermediate- and high-grade endometrial cancer staging. JAMA Surg..

[15] Marchocki Z., Cusimano M.C., Clarfield L., Kim S.R., Fazelzad R., Espin-Garcia O., Bouchard-Fortier G., Rossi E.C., Stewart K.I., Soliman P.T. (2021). Sentinel lymph node biopsy in high-grade endometrial cancer: A systematic review and meta-analysis of performance characteristics. Am. J. Obstet. Gynecol..

[16] Zhang S., Zheng J., Zhu D. (2025). Long-term outcomes in patients with endometrial cancer after sentinel lymph node biopsy versus lymphadenectomy alone: A meta-analysis. Front. Oncol..

[17] Gu Y., Cheng H., Zong L., Kong Y., Xiang Y. (2021). Operative and oncological outcomes comparing sentinel node mapping and systematic lymphadenectomy in endometrial cancer staging: Meta-analysis with trial sequential analysis. Front. Oncol..

[18] Garrett A.A., Wield A., Mumford B., Janmey I., Wang L., Grosse P., MacArthur E., Buckanovich R., Courtney-Brooks M., Sukumvanich P. (2022). Clinical factors associated with failed sentinel lymph node mapping in endometrial cancer. Gynecol. Oncol. Rep..

[19] Khoury-Collado F., St. Clair C., Abu-Rustum N.R. (2016). Sentinel lymph node mapping in endometrial cancer: An update. Oncologist.

[20] Bodurtha Smith A.J., Fader A.N., Tanner E.J. (2017). Sentinel lymph node assessment in endometrial cancer: A systematic review and meta-analysis. Am. J. Obstet. Gynecol..

[21] Cianci S., Rosati A., Vargiu V., Capozzi V.A., Sozzi G., Gioè A., Alletti S.G., Ercoli A., Cosentino F., Berretta R. (2021). Sentinel lymph node in aged endometrial cancer patients “The SAGE Study”: A multicenter experience. Front. Oncol..

[22] Papageorgiou D., Zachariou E., Sapantzoglou I., Tsakos E., Xydias E.M., Dimitroulis D., Plevris N. (2025). Impact of body mass index on robotic surgery outcomes in early-stage endometrial cancer: A retrospective cohort study. Cancers.

[23] Fu H., Zhang J., Zhao S., He N. (2023). Survival outcomes of robotic-assisted laparoscopy versus conventional laparoscopy and laparotomy for endometrial cancer: A systematic review and meta-analysis. Gynecol. Oncol..

[24] Walker J.L., Piedmonte M.R., Spirtos N.M., Eisenkop S.M., Schlaerth J.B., Mannel R.S., Barakat R., Pearl M.L., Sharma S.K. (2012). Recurrence and survival after random assignment to laparoscopy versus laparotomy for comprehensive surgical staging of uterine cancer: Gynecologic Oncology Group LAP2 Study. J. Clin. Oncol..

[25] Raffone A., Fanfani F., Raimondo D., Rovero G., Renzulli F., Travaglino A., De Laurentiis U., Santoro A., Zannoni G.F., Casadio P. (2023). Predictive factors of sentinel lymph node failed mapping in endometrial carcinoma patients: A systematic review and meta-analysis. Int. J. Gynecol. Cancer.

[26] Sozzi G., Fanfani F., Berretta R., Capozzi V.A., Uccella S., Buono N., Giallombardo V., Di Donna M.C., Monterossi G., Restaino S. (2020). Laparoscopic sentinel node mapping with intracervical indocyanine green injection for endometrial cancer: The SENTIFAIL study, a multicentric analysis of predictors of failed mapping. Int. J. Gynecol. Cancer.

[27] Taliento C., Scutiero G., Cucinella G., Chiantera V., Pontrelli G., Klaric M., Belci D., Steinkasserer M., Restaino S., Martinello R. (2025). Predictive factors for failed sentinel lymph node mapping in endometrial cancer: A retrospective multicenter study. J. Surg. Oncol..

[28] Frumovitz M., Plante M., Lee P.S., Sandadi S., Lilja J.F., Escobar P.F., Gien L.T., Urbauer D.L., Abu-Rustum N.R. (2018). Near-infrared fluorescence for detection of sentinel lymph nodes in women with cervical and uterine cancers (FILM): A randomised, phase 3, multicentre, non-inferiority trial. Lancet Oncol..

[29] Ruscito I., Gasparri M.L., Braicu E.I., Bellati F., Raio L., Sehouli J., Mueller M., Panici P.B., Papadia A. (2016). Sentinel node mapping in cervical and endometrial cancer: Indocyanine green versus other conventional dyes—A meta-analysis. Ann. Surg. Oncol..

[30] Dai Z.Y., Shen C., Mi X.Q., Pu Q. (2023). The primary application of indocyanine green fluorescence imaging in surgical oncology. Front. Surg..

